# A qualitative exploration of factors influencing medical staffs’ decision-making around nutrition prescription after colorectal surgery

**DOI:** 10.1186/s12913-019-4011-7

**Published:** 2019-03-19

**Authors:** Megan Rattray, Shelley Roberts, Ben Desbrow, Martin Wullschleger, Tayla Robertson, Ingrid Hickman, Andrea P. Marshall

**Affiliations:** 1 School of Allied Health Sciences, Griffith University, Gold Coast Campus, QLD 4222 Australia; 2 School of Allied Health Sciences, Griffith University and Gold Coast Hospital and Health Service, Griffith, QLD 4222 Australia; 3 Department of Trauma, Gold Coast University Hospital, Gold Coast, 4222 Australia; 4 Department of Nutrition and Dietetics, Princess Alexandra Hospital, Gold Coast, QLD Australia; 5 Department of Nutrition and Dietetics, Princess Alexandra Hospital and Mater Research Institute, University of Queensland, Gold Coast, QLD Australia; 6 Menzies Health Institute Queensland, Griffith University and Gold Coast Health, Gold Coast, 4222 Australia

**Keywords:** Early oral feeding, Perioperative nutrition care, Postoperative nutrition care, Enhanced recovery after surgery

## Abstract

**Background:**

Enhanced Recovery After Surgery (ERAS) guidelines recommend early oral feeding with nutritionally adequate diets after surgery. However, studies have demonstrated variations in practice and poor adherence to these recommendations among patients who have undergone colorectal surgery. Given doctors are responsible for prescribing patients’ diets after surgery, this study explored factors which influenced medical staffs’ decision-making regarding postoperative nutrition prescription to identify potential behaviour change interventions.

**Methods:**

This qualitative study involved one-on-one, semi-structured interviews with medical staff involved in prescribing nutrition for patients following colorectal surgery across two tertiary teaching hospitals. Purposive sampling was used to recruit participants with varying years of clinical experience. The Theoretical Domains Framework (TDF) underpinned the development of a semi-structured interview guide. Interviews were audio recorded, with data transcribed verbatim before being thematically analysed. Emergent themes and sub-themes were discussed by all investigators to ensure consensus of interpretation.

**Results:**

Twenty-one medical staff were interviewed, including nine consultants, three fellows, four surgical trainees and five junior medical doctors. Three overarching themes emerged from the data: (i) Prescription preferences are influenced by perceptions, experience and training; (ii) Modifying prescription practices to align with patient-related factors; and (iii) Peers influence prescription behaviours and attitudes towards nutrition.

**Conclusions:**

Individual beliefs, patient-related factors and the social influence of peers (particularly seniors) appeared to strongly influence medical staffs’ decision-making regarding postoperative nutrition prescription. As such, a multi-faceted approach to behaviour change is required to target individual and organisational barriers to enacting evidence-based feeding recommendations.

**Electronic supplementary material:**

The online version of this article (10.1186/s12913-019-4011-7) contains supplementary material, which is available to authorized users.

## Background

Enhanced Recovery After Surgery (ERAS) guidelines assist clinicians in making informed decisions regarding optimal perioperative care among colorectal patients [1, 2]. Early oral feeding (EOF), defined as initiating liquids and solids within 24 h after colorectal surgery [1–3], is an essential component of ERAS that has proven to be safe and beneficial [4, 5]. However, recent studies have demonstrated variable and often poor adherence to EOF recommendations and frequent use of nutritionally inadequate diets (i.e. clear liquids) among patients that undergo gastrointestinal surgery [6–8]. In addition to missing out on the benefits associated with EOF, delayed feeding and extended use of liquid only diets can increase the risk of developing or exacerbating protein-energy malnutrition (PEM) [9]. Among surgical patients, PEM has been associated with increased incidence of complications [10], longer lengths of stay [11, 12] and higher rates of hospital readmission [11]. As such, efforts to improve postoperative nutrition prescription practices are needed to optimize patient and healthcare outcomes.

Translating evidence-based guidelines into practice often requires a multifaceted approach, including education, training, and behaviour change techniques [13]. However, an important aspect of translational work is to understand the perceptions of those who will be involved and the environment where the change will occur before attempting to enact change [14]. Medical staff are influential decision makers of any ERAS program and are responsible for prescribing patients’ diets after surgery. As such, previous studies have explored surgeons’ knowledge, attitudes, and behaviours towards perioperative nutritional practices [15–17]. While this work has improved our understanding of the gaps between nutrition-related knowledge, attitudes, and practices [15–17], these studies have mainly focused on preoperative nutrition-related components, and/or have employed an atheoretical design. Given the complexity of the clinical environment, it is important to employ empirically-supported theories to understand staffs’ behaviour. As such, establishing a better theoretical understanding of doctors’ decision-making is required to inform development of targeted strategies to improve postoperative nutrition practices among patients who have undergone colorectal surgery.

The Theoretical Domains Framework (TDF) is a theoretically-based approach to behaviour change. It consists of 14 domains that explain human behaviour in complex clinical environments: behavioural regulation; optimism; beliefs about consequences; skills; social/professional role and identity; emotion; beliefs about capabilities; goals; memory, attention and decision processes; reinforcement; intentions; environmental context and resources; social influence; and knowledge [18]. Consequently, this framework has been utilised in previous work to detect barriers and enablers to evidence-based guideline use among clinicans, and consequently inform interventions to increase uptake of evidence into habitual practice [19]. As such, the aim of this study was to use the TDF to explore the factors influencing medical staffs’ decision-making regarding postoperative nutrition prescription among non-critically ill colorectal patients.

## Methods

### Study design and setting

This study was conducted in a constructivist–interpretivist paradigm using semi-structured interviews, underpinned by the TDF [18], to explore decision-making of surgeons regarding the prescription of nutrition after colorectal surgery. Constructivists consider that meaning is discovered through exploration of a phenomenon within its context and through their interactions with the data, hence this approach was considered the most appropriate [20]. Interviews were conducted across two tertiary teaching hospitals in Queensland, Australia. Approximately 180–200 colorectal resections are performed at each site per year. No ERAS or standardized perioperative nutrition care protocol were formally implemented at either hospital at the time of data collection; rather, medical staff prescribed feeding and other postsurgical orders at their own discretion. Ethical approval was gained by the relevant Hospital and University Human Research Ethics Committees (reference numbers: HREC/17/QGC/101 and GUREF/2017/389). The methodology of this study was devised per the Consolidated Criteria for Reporting Qualitative Research (COREQ) [21].

### Participants and recruitment

Any medical staff involved in prescribing diets for colorectal patients in the postoperative period were eligible to participate. With assistance from the nurse unit manager, clinical facilitator and or clinical nurse consultant, potential participants meeting the inclusion criteria were identified at each site. Eligible participants were approached by one of the interviewers (MR or TR) and invited to participate. Purposive sampling was used to recruit a mixture of participants in terms of position and years of clinical experience. We used the concept of data saturation to determine when to cease recruitment. In short, we conducted interviews at both sites until no new information was being offered (i.e. when the interviewers begun to hear the same comments again and again). This occurred after seven and ten interviews; however, an additional two staff were subsequently interviewed at both sites to confirm that data saturation had, indeed, occurred. Informed consent was obtained from all participants prior to initiating interviews.

### Data collection

The TDF underpinned the development of a semi-structured interview guide (Additional file 1). The research team constructed around two to five questions within each domain. The interview guide was piloted among clinicians (who were not participants of the study) prior to data collection, resulting in minor changes to wording and the formation of new questions. A conversational style of interviewing was adopted with the semi-structured interview guide and staff responses providing direction for the interviewers (MR and TR). The two interviewers, who had a background in dietetics and were trained in interview techniques, interviewed all participants between June 2017 and January 2018. One interviewer was the ward dietitian who had an established working relationship with a number of staff prior to interviews. The second interviewer had no established relationships with participants prior to interviews. All staff were interviewed one-on-one at a time and place convenient to them. Interviews lasted between 11 and 39 min (average 24 min) for a total of 510 min of data. Interviews were audio recorded and later transcribed verbatim for analysis.

### Data analysis

All interviews were transcribed verbatim. Braun & Clarke’s 6-step guide [22] was used to thematically analyze interview data and identify emerging themes. This process involved the lead author (MR) reading and rereading transcripts for immersion in the data, highlighting key quotes and then developing codes based on participants’ verbatim statements. Codes were grouped then into sub-themes and themes based on common threads throughout the data. The TDF was then applied to categorise subthemes into the relevant theoretical domains. Data from each hospital were analysed separately prior to being combined. An electronic audit trail of this process was reviewed by a second researcher (SR) to ensure consistent interpretation of the data and organisation of codes. Any disagreements or contested themes/ subthemes/ theoretical domains were discussed between the two researchers until consensus was reached. The analysis was an iterative process, with the two researchers constantly referring back to the raw data to substantiate emerging ideas and themes.

Methodological rigour and trustworthiness was upheld using several strategies. The interview guide was piloted among seven clinicians, which resulted in minor changes to wording. Reflexivity occurred through the interviewers: i) completing contact summary forms after each interview to enhance future interviews; and ii) documenting their preconceptions and ideas throughout the analysis process to consider how this influenced their approach to interviews and subsequent analysis. Trustworthiness of data was enhanced through frequent discussions among the research team to ensure codes, sub-themes and themes adequately described and encompassed the data. Further, the multidisciplinary nature of the research team, consisting of clinicians and/or researchers from medical, dietetic and nursing backgrounds, provided a range of insights into data analysis and interpretation.

## Results

A total of 21 medical staff were interviewed, including nine colorectal surgical consultants, three surgical fellows, four surgical trainees (two registrars and two principle house officers), and five junior medical doctors (one junior house officer and four interns). Nine and twelve staff were recruited at site one (S1) and site two (S2), respectively. No staff members who were approached declined to participate. Participant demographics are outlined in Table 1 and the medical career structure outlining the description of each position is depicted in Additional file 2. Approximately three-quarters of participants were male (*n* = 16). Through our analysis, we identified three themes and various subthemes which appeared to influence medical staffs’ decision-making. These are depicted in Table 2 and described in detail below. Of note, the use of “many” or “most” throughout this section is equivalent to ~ 75% or more of the sample in question.

**Table 1:** Participant demographics

Site	Position	Age
1	Senior registrar	41–45
1	Surgical intern	26–30
1	Consultant	46–50
1	Consultant	46–50
1	Surgical fellow	41–45
1	Consultant	41–45
1	Surgical intern	26–30
1	Principal House Officer	26–30
1	Surgical fellow	36–40
2	Consultant	51–55
2	Consultant	46–40
2	Registrar	31–35
2	Surgical resident	21–25
2	Consultant	41–45
2	Surgical resident	21–25
2	Consultant	41–45
2	Consultant	46–50
2	Consultant	51–55
2	Surgical Fellow	36–40
2	Junior House Officer	26–30
2	Principal House Officer	26–30

**Table 2:** Themes and subthemes

Themes	Subthemes
(1) Prescription preferences are influenced by perceptions, experience and training	a. Perceived risk-benefit ratio
	b. Training and past behaviours
	c. Positive and negative experiences
(2) Modifying prescription practices to align with patient-related factors	a. Considering surgical factors and patient demographics
	b. Progressing feeding in line with patients’ clinical status
	c. Meeting patients’ expectations and needs
(3) Peers influence prescription behaviours and attitudes towards nutrition	a. Social influence on prescription behaviours
	b. Social influence on attitudes towards nutrition

### Prescription preferences are influenced by perceptions, experience and training

Overall, the majority of surgeons (colorectal consultants, fellows, and surgical trainees) said they would usually prescribe liquids on the day of surgery or the following morning if patients returned from theatre in the evening. However, surgeons’ responses varied regarding when they usually or would ideally prescribe solids after surgery. Around half said they would usually or ideally prescribe liquids and solids within 24 h after surgery and thus were defined as ‘EOF advocates’. The remaining surgeons spoke of usually commencing solids within 48–72 h after surgery. Concerning diet type, most surgeons indicated they preferred prescribing free fluids over clear fluids immediately after surgery for reasons such as greater palatability and higher nutritional value. Further, most consultants, fellows, and surgical trainees indicated that the habitual use of clear fluids in the postoperative setting was scientifically unfounded. Most junior medical doctors believed clear fluids should be used prior to free fluids as they are more “*easily tolerated*” and help with “*bowel rest*”. However, one junior medical doctor did express scepticism regarding the “*science behind*” using clear fluids over free fluids following surgery. The majority of junior medical doctors were also uncertain of the safety for patients recommencing solids within 24 h after surgery.

Staff responses towards EOF appeared to be influenced by: 1) their perception of the risk-benefit ratio, 2) training and past behaviours, and 3) positive and negative experiences. Representative quotations for these sub-themes and the TDF domains they align with are presented in Table 3.

**Table 3:** Representative quotations for theme 1: Prescription preferences are influenced by perceptions, experience, training and guidelines

Subtheme	TDF domain	Quotes
Perceived risk-benefit ratio	Beliefs about consequences/ Motivation and goals	*“Early oral feeding is associated with a significant reduction specifically in septic or infective complications. There is a 20% increase in nausea and vomiting, however, but overall, the rate of postoperative ileus is actually decreased by early oral feeding and not increased”* [P01: Consultant] *“Early post-op feeding is important… Probably the biggest study that was done was a meta-analysis that looked at I think… 14 or 15 studies, and there wasn’t any significant findings but there was certainly a trend towards a lot of reduced complications… so reduced anastomotic leak rate, reduced intra-abdominal abscesses, a trend towards early return of gut function… so they’re all pretty important things, and there was certainly no increase in complications…I mean whether it does any good…hard to know, the evidence is not strong…but it probably does. It certainly doesn’t do any harm… I guess it’s got other protective things like gut mucosa, and all of those sorts of things… so, you know… early oral feeding is important”* [P03: Fellow] *“Nutrition, of course, is very important… but once the bowel starts working, once the bowel activity starts and the bowel continuity is ensured – once we are sure the anastomosis is intact, they should be going back to their normal diet. That is what we would like to see”* [P14: Consultant] *“I would be scared commencing solids within 24 h in a lot of these patients who have this fresh join which could leak into the bowel [abdomen]”* [P13: Intern]
Training and past behaviours	Nature of the behaviour/ Motivation and goals/ Skills	*“I’m sure the guys who are very reluctant to start oral feeding early is just because that was the way they were taught and they haven’t had any issues with it, so why would they change. Whereas, I was taught, nah it’s ok to do that, and you’ve just got to be aware of if they’re heading in the wrong direction, and when to cut back on their oral intake”* [P10: Consultant] *“I suppose I have been exposed to the earlier practices where people were… fasted for a week...when I first started training. But as I became more senior I was exposed to more ERAS protocols in sub-speciality training with earlier feeding... so I was introduced to the concept of earlier feeding”* [P15: Consultant] *“What I’ve seen is that there is definitely a change in the school of thought. I’m a junior registrar so I’m in the area that is quite different [to what it was previously]. The older surgeons are much more hold-offish. But the younger and more developing surgeons are...and definitely the fellows – they defiantly seem to be more on the front foot with nutrition after surgery”* [P11: Principal House Officer] *“I’m surprised to hear that guidelines recommend commencing solid feeding within 24 h after surgery among lower gastrointestinal patients. That is interesting…because if on ward rounds the consultant the next day after surgery was like, ‘full diet’ I would be like ‘whoa, are you sure?’ Like, I would double check. Yeah, so that is surprising”* [P13: Intern] (NB: This was in response to the interviewer sharing guideline recommendations with the participant)
Positive and negative experiences	Beliefs about consequences/ Nature of the behaviours/ Motivation and goals	*“I was the clinical lead of the Enhanced Recovery After Surgery Program…we recorded data before the implementation of ERAS. There was quite a substantial reduction in morbidity and length of stay associated with the implementation of ERAS* [P01: Consultant] *“Earlier this year, we had a few patients that [*sic*] had postoperative vomiting and had aspirations ... aspiration pneumonia and so from that there was a bit of a reflex action to recommend that we don’t push people’s oral intake as much ... [so there was] kind of reaction to those couple of complications”* [P09: Fellow]

*TDF* Theoretical domains framework

#### Perceived risk-benefit ratio

Staffs’ attitudes towards postoperative nutrition appeared to be profoundly shaped by their perceptions of the risk-benefit ratio of receiving EOF. There was a strong contrast in responses between those who were ‘EOF advocates’ and those who were not. Most ‘EOF advocates’ believed recommencing liquids and solids within 24 h after surgery was safe, and many highlighted the potential advantages associated with EOF, such as faster return of gut function, reduced risk of complications and shorter lengths of stay. It appeared these staff believed the potential benefits associated with EOF outweighed the potential risks or at least, held the belief that EOF was safe (e.g. didn’t increase the risk of an anastomotic leak). Alternatively, the surgeons who were *not* ‘EOF advocates’ were less likely to acknowledge the potential benefits of EOF, but instead spoke of the importance of safety (e.g. ensuring the anastomosis remained intact and preventing aspiration) over the provision of adequate nutrition. This belief was reflected in the majority of junior medical doctor responses.

#### Training and past behaviours

Staffs’ training and past behaviours appeared to contribute to their acceptability of EOF. Many ‘EOF advocates’ spoke of how their training had influenced their dietary prescription habits and commented on how this may explain the behaviours of their peers. For example, the surgical trainees, fellows and consultants who were defined as ‘EOF advocates’ explained that evidence for ERAS and EOF existed during their medical or sub-specialty training, which appeared to increase their confidence in its safety and benefits. In fact, one consultant suggested the reason some surgeons remain “*reluctant*” to prescribe EOF is due to training under the ‘old’ feeding paradigm. Indeed, a consultant who was *not* defined as an ‘EOF advocate’ stipulated that they have been feeding the same way for a “*long time now*” and expressed scepticism over whether changing practice would affect patients’ “*long term outcomes*”. All surgical trainees, fellows and consultants were confident in their skills and capabilities to judge when and what to prescribe patients after surgery. Many junior medical doctors were unfamiliar with ERAS/EOF guidelines or even these concepts, despite approaching the end of their rotation and were not confident in their ability to judge when patients were ready for liquid and solid feeding.

#### Positive and negative experiences

Differences in attitudes towards EOF also appeared to be influenced by staffs’ negative or positive experiences with it. For example, several ‘EOF advocates’ had experience with formalised ERAS protocols in other hospitals, which appeared to increase their confidence in the safety and effectiveness of EOF. Alternatively, a fellow spoke of an incident that occurred at one of the study sites, where a patient acquired aspiration pneumonia from vomiting after EOF, which decreased surgeons’ confidence in re-introducing solids early in the postoperative period.

### Modifying prescription practices to align with patient-related factors

Many staff spoke about making nutrition care decisions in the *“patient’s best interest”*, which involved adapting their ‘usual feeding practices’ to align with patients’ procedural factors and demographics; clinical status; and preferences and needs. Of note, decisions around what was best for patients were heavily influenced by staffs’ beliefs (discussed in theme 1). Quotations to substantiate these sub-themes and the TDF domains they align with are presented in Table 4.

**Table 4:** Representative quotations for theme 2: Modifying prescription practices to align with patient-related factors

Subtheme	TDF domain	Quotes
Considering surgical factors and patient demographics	Knowledge/ Behavioural regulation/ Belief about consequences	“*We used to use clear fluids years ago, but not anymore – only in cases where there might be an ileus then we introduce that step*” [P17: Consultant] “*There are some conditions where we do delay the feeding deliberately depending on the amount of adhesions we have intraoperatively, but even then a small amount of feed is usually useful. The way I deal with it is I usually give a small amount of clear fluids*” [P06: Consultant] *“Sometimes with elderly patients...[for a] lady who’s day one post right hemi... you sort of say ‘oh, should I just leave her on free fluids today, or no it doesn’t matter - it’s good for her to eat and get going?’. The risk for her is that if you upgrade things, and she gets distended, and she vomits; she might then go backward...because once someone vomits and someone puts a nasogastric tube in, then it’s clear fluids for a while…so it might set her back 48–72 h if you do upgrade things too early in an elderly patient”* [P03: Fellow] “*I would be happy to prescribe ERAS to all patients. The only privy I make to that is the very elderly or the very frail...I titrate their feeding to their gut function a lot more*” [P16: Fellow]
Progressing feeding in line with patients’ clinical status	Knowledge/ Behavioural regulation	“*The next morning [POD1]… depending on how they went [on clear fluids overnight], if they had any nausea or vomiting, then they stay on clear fluids… but if they were feeling fine, then they’d slowly upgrade to free [fluids], and if they’ve started opening their bowels and they’re tolerating free fluid that’s when we start upgrading them to a full solid die*t” [P04: Junior House Officer] *“Quite often the patients request it themselves [to be upgraded]; they get sick of it [a fluid diet], so they’re asking for food”* [P02: Intern] “*I commence earlier feeding. I would start normally from day one [night of surgery] …on free fluids and then if it is tolerated move them onto a light diet [usually the next day]…… I do not rely so much on bowel opening or bowel sounds – I find that is not as reliable*” [P15: Consultant]
Meeting patients’ expectations and needs	Memory, attention and decision processes	*“[Postoperative feeding] should be patient driven really, that’s the point I’m trying to make, is that the patient actually knows best what they’re ready for… saying ‘you can have whatever you feel like’…that’s probably the best thing. At the moment they’re not. I mean many patients are told, “you can have this, and then you can have that*” [P01: Consultant] *“There might be a patient who feels a little bit anxious about eating and they say they want to stay on the free fluids for an extra day. In that kind of situation, I would probably let them have that because if we give them solids their anxiety might mean they eat less and therefore they are not reaching their nutritional requirements*” [P09: Fellow] “*No [I don’t think patients should be involved in decisions regarding their dietary status]…Ah, well that’s unfair. Some of them are probably sensible enough, but some of them have no idea what we’ve done, and I guess if I’m doing 200 bowel resections a year, I’m probably more experienced than they are despite their five hours on the internet researching what they think they should be doing*” [P20: Consultant] “*I don’t think they really are actually [involved in their diet-related decisions]. Even when they say ‘oh you know I’m really hungry’…often…oh not so much this team actually, they’re pretty good…but it’s still not viewed as a partnership thing, it’s very much the team…the fellow will say ‘yes’ or ‘no’”* [P19: Intern]

*TDF* Theoretical domains framework

#### Considering surgical factors and patient demographics

Considering patients’ surgical factors and demographics when determining the type and time of feeding were discussed by the majority of staff. While most surgeons said they preferred free fluids over clear fluids in usual practice, there were certain circumstances in which they would prescribe clear fluids (e.g. among patients at high risk of developing an ileus or gastroparesis). In these instances, clear fluids would be used as a “trial run” or so the medical team could “deal with the complication, not their diet”. Further, two consultants spoke of using clear fluids when a nasogastric tube (NGT) was in place, believing clear fluids as opposed to free fluids would “*come up the NGT*” more freely. Lastly, several surgeons stated they would only use clear fluids if they anticipated that a patient would need a subsequent operation.

The majority of surgeons said they would delay commencing solids among patients who underwent an emergent procedure due to swelling and dilation. Postponing solid intake among patients who underwent a small bowel anastomosis or stoma was also discussed, where staff expressed the need to wait “*two days for the swelling at the site to open up”* before prescribing a full diet. In fact, one consultant spoke of prescribing a low fibre diet among these patients to ensure food residue *“doesn’t block up the small bowel anastomosis or stoma”*. Many staff also discussed *“going slower”* among patients who underwent a right hemicolectomy, stating they are at a greater risk of developing an ileus. However, this criterion was often considered in conjunction with other ‘risk factors’ such as older age. Further, older age for some surgeons was a singular risk factor in which they would alter their usual prescription habits through titrating “*their feeding to their gut function*”. It appeared, in these circumstances, staff were concerned that prematurely upgrading patients to a solid diet could result in adverse outcomes (e.g. vomiting and aspiration) and thus delay the patient’s recovery.

#### Progressing feeding in line with patients’ clinical status

Using clinical indicators to determine diet progression on a day-to-day basis were discussed by all staff. Many surgeons spoke of progressing patients’ dietary status once they were “*tolerating*” the former diet, which was assessed during daily ward rounds. However, the term “*tolerating*” was defined differently by staff. The absence of postoperative nausea and vomiting and abdominal distension were common criterion raised by all staff to determine if patients were ready to progress to the next diet. Several consultants also spoke of how passing flatus may contribute to their decision regarding when a patient was ready to commence solids, while other consultants explicitly stated that they find this marker “*unreliable*”. Interestingly, the majority of junior medical doctors believed that passing flatus or a bowel motion were common markers used to upgrade a patients’ dietary status. Further, many junior staff, particularly at S2, said hunger or boredom of choice was a common reason why patients were upgraded from liquids to solids.

#### Meeting patients’ expectations and needs

Most staff said they were accepting of patients participating in their nutrition care decisions. However, the level of involvement patients should have in their decisions varied from staff-to-staff. Several surgeons spoke of how prescribing an unrestricted diet from postoperative day one (POD1) promotes patient participation in care as it enables patient-directed feeding, being that patients “*can have whatever they want” rather than ‘this is what the doctor said you should eat and you should eat it*’. Other surgeons, however, viewed decision making as more of a partnership, whereby they would factor in patients’ preferences or concerns when determining their dietary status, which in some cases resulted in patients staying on fluids for longer due to their “*anxiety*” surrounding reintroducing solids. Generally, these two examples of patient participation in care were expressed among EOF advocates. Alternatively, several surgeons stated they were better positioned to determine a patient’s dietary status, expressing scepticism of involving patients in decisions around their postoperative nutrition. Several surgeons also spoke of how educating and encouraging patients preoperatively or postoperatively about nutrition was a demonstration of involving them in their care. Most junior doctors and several surgeons explicitly stated they did not think patients were involved in nutrition care decisions, rather they were “*told what to do*”.

### Peers influence prescription behaviours and attitudes towards nutrition

The direct and indirect influence of peers on individual doctors’ decision-making regarding postoperative feeding was evident. Representative quotations for these subthemes and the TDF domains they align with are presented in Table 5.

**Table 5:** Representative quotations for theme 3: Peers influence prescription behaviours and attitudes towards nutrition

Subtheme	TDF domain	Quotes
Social influence on prescription behaviours	Social/ Professional role and identify/ Emotion	*“I don’t like clear fluids because I think they taste terrible and it doesn’t [do anything]. The difficulty here is that some of the bosses do like clear fluids initially, so I have to follow what they do. But my preference would be to give free fluids immediately, rather than clear*” [P12: Senior registrar] *“So I make the plan when I’m around and then I say to the boss, ‘I’m changing this diet’…sometimes they will say things like, ‘I think that is a bit quick, but I’m sure we will see’. But at the end of the day they usually just say ‘ok’”* [P11: Principal House Officer] *“Teams on the wards are run by junior staff so they sometimes often have different ideas... [it] is something that is out of my control because I am not on the ward every day. We [consultants] suggest what to do, but sometimes they [junior staff] feel that the patient is blowing up [distended] [so] they will delay by one day. But there are sometimes changes in what I normally do because of different people on the wards*” [P17: Consultant]
Social influence on attitudes towards nutrition	Social/ Professional role and identify/ Environment context and resources	*“I wasn’t made to feel like it [nutrition] was important…the things that I knew were really important were picking up on if things were not going to plan, so complications such as a leak or a massive infection and things that are going to kill them… It was not a priority for us at all…and I definitely wasn’t made aware of it in like, when we do consultant rounds, there was never any focus on feeding*” [P13: Intern]. *“I have learnt that people can eat sooner than I thought they could after surgery. My bosses have said ‘don’t bother, like, doing graduated things, just change them over. They are fine. They can eat and drink’. I just think most of my bosses want people eating as soon as possible*” [P18: Intern]*.* “*Well for me [the importance of nutrition] is personally reasonably high. Nutrition is critical to health and surgical outcomes. But I feel like it is not hugely valued within our profession. I think it is very undervalued by surgeons – the role of nutrition…and I think they may reflect in junior staff in terms of attitude and things as well. But surgeons themselves, I think are really...generally poor advocates for good quality nutrition and they may or may not eat well for themselves and they may or may not know much about nutrition themselves*” [P16: Fellow]

*TDF* Theoretical domains framework

#### Social influence on prescription behaviours

While all surgeons discussed their usual preferences for nutrition after surgery, many spoke of how their prescribing habits were directly influenced by their peers. The hierarchical influence of peers was particularly evident. For example, the majority of surgical trainees and fellows spoke of how the consultant they worked under directly influenced their prescribing; i.e. they prescribed clear over free fluids to align with their consultants’ preference. However, this generally referred to prescribing the initial diet, which was often determined in theatre. Dietary upgrades thereafter were principally determined by the colorectal fellow or surgical trainee leading the ward round; however, their decisions were often discussed with the attending consultant. In fact, one surgical trainee discussed how the presence of a colorectal fellow who was an ‘EOF advocate’ could strongly influence practice on the ward, as patients were more likely to be upgraded to solids early after surgery. Alternatively, a surgical trainee discussed how some fellows or surgical trainees may “*drag their feet in upgrading a patient’s diet in fear of it looking bad the next day*”. Interestingly, a consultant spoke of changing the way he would usually prescribe nutrition based on who was present on the ward.

#### Social influence on attitudes towards nutrition

Peers indirectly influenced postoperative dietary prescription practices with their attitudes towards nutrition. One fellow thought nutrition was *“undervalued”* among surgeons, which was reflected in *“junior staffs’ attitudes”*; this was evident from junior staffs’ responses. When EOF was not reinforced by seniors, junior medical doctors did not value the role of nutrition in the postoperative period. For example, one intern stated that they were not “*made to feel like*” nutrition was important, particularly during consultant rounds; rather, the priority was “*picking up complications – things that are going to kill them*”. Alternatively, some junior doctors perceived nutrition in the postoperative recovery as important because their seniors emphasised it: “*my bosses want people eating as soon as possible*”. Interestingly, while some staff thought it was their “*job*” to teach junior staff, a handful did express concern regarding the lack of awareness of ERAS and EOF among junior medical doctors.

## Discussion

This study explored factors influencing surgeons’ decision-making regarding the prescription of nutrition following colorectal surgery. Individual beliefs, patient-related factors and the social influence of peers were key factors that appeared to strongly influence staffs’ decision-making. These findings may be used to inform the development of educational and behaviour change strategies to improve postoperative nutrition prescription practices.

Surgeons held varied views on when they would usually or ideally prescribe nutrition after colorectal surgery. While most consultants, fellows and surgical trainees described prescribing fluids early in the postoperative period, only half said they would usually or ideally prescribe solids within 24 h after surgery. Behaviour change theories propose that people’s beliefs about the likely outcomes of an innovation and the value attached to those outcomes heavily influences their likelihood of adoption [23]. Indeed, surgeons’ perceptions of the risk-benefit-ratio of EOF appeared to influence prescription preferences in this study. Surgeons who preferred early solids and fluids (i.e. EOF advocates) believed this practice was safe and thought the benefits, such as improved return of gut function and reduced postoperative complications, infections and length of stay [4, 5] outweighed potential risks. Alternatively, staff hesitant of EOF consistently reiterated the risks of EOF and did not appear to view the practice as particularly advantageous. Collectively, these findings suggest that in order for surgeons to prescribe both early liquids and solids after colorectal surgery, they need to believe there is a relative advantage to this practice and that it is indeed safe.

Differences in surgeons’ perceptions of EOF are likely explained by their training and past experiences; findings consistent with previous studies exploring factors predicting adoption of evidence-based practice [24, 25]. Many EOF advocates discussed how ERAS had been at the forefront of their training and some had been exposed to formalised protocols implemented in other hospitals. This appeared to increase their confidence and knowledge in prescribing early fluids and solids. Alternatively, most surgeons who were hesitant of EOF were trained under the old feeding paradigm, where resumption of nutrition was indicated only after recovery of bowel function; a belief which remains entrenched as evidenced by surgeons’ responses. Changing long-standing attitudes and behavior of clinicians can be difficult [26]. Previous work shows education can increase knowledge, however may not immediately improve practice [27]. Rather, changing behavior is a continuous process whereby evidence-based innovations may undergo a lengthy period of negotiation among adopters, in which their meaning is discussed, contested, and reframed [28]. Such deliberation can increase or decrease the potential adopter’s perceived relative advantage of the innovation [29]. Indeed, this appeared to be evident within the current study, when a surgeon spoke of a negative EOF experience that occurred at one of the sites which decreased hesitant staff members’ confidence in prescribing early solids after colorectal surgery. For this reason, it is important to provide education and training on the benefits and safety of EOF, while also providing reassurance (e.g. through regular monitoring and feedback to staff) in the early stages of adoption to facilitate changes in surgeons’ prescription practices.

Behaviour change theories [23] recognise the role of the social environment in influencing behaviour. Individuals’ decision-making were directly and indirectly influenced by their seniors in the current study; a finding consistent with previous work [30]. The inter-professional influence of the attending consultant was evident, as fellows and surgical trainees expressed they prescribed diets in line with the consultant’s preferences, despite holding alternative views. For example, while most surgeons (particularly fellows and surgical trainees) specified their preference for using free fluids directly after surgery, clear fluids were still habitually prescribed at both hospitals as some consultants held this preference. The idea that doctors are expected not to question decisions of more senior staff has been identified in previous work [31]. These findings suggest that for EOF practices to align with evidence-based guidelines, behavioural change strategies particularly need to target consultants and fellows, given their direct influence on other staff. Further, changing behaviours and attitudes of senior staff towards EOF and nutrition in general will likely influence attitudes and perceptions of junior staff, considering surgeons are trained in highly hierarchical structures where practitioners tend to avidly follow the lead of their seniors; a finding demonstrated in the current study and previous work [32].

Lastly, patient-related factors, such as procedure type, demographics and clinical status appeared to strongly influence clinicians’ decision-making. Many surgeons described delaying the prescription of solids among patients who underwent procedures involving the small bowel or ascending colon due to “swelling” and “increased risk of ileus”. Indeed, evidence suggests there is a higher incidence of paralytic ileus after right-sided ileo-colic anastomosis compared with left-sided colo-colic and colo-rectal anastomosis [33, 34]. Further, in a recent study the safety of patients who underwent an ileostomy closure to have a full diet on POD1 were reported. However, this approach did result in a high incidence of vomiting (45%), which the authors hypothesised was due to oedema and spasm at the site of the anastomosis [35]. Hence, feeding recommendations may require adaptation for procedures involving the small bowel [35], particularly among elderly patients [33], who appear to be commonly considered as high-risk by surgeons. However, not all patient-related factors that influenced surgeons’ decision-making were evidence-based. For example, some staff spoke of using clear fluids over free fluids, soft or low fibre diets over full diet, and progressing patients’ diet types based on their bowel function; practices that are scientifically unfounded. Lastly, while many surgeons spoke of making nutrition care decisions in the “patient’s best interest”, this view was often held from their perspective and not in consultation with the patient. In fact, only a minority of surgeons described factoring patients’ food preferences and nausea or hunger ratings into their decision-making, while several stated they were better positioned to determine patients’ dietary needs. However, when patients participate in their care they have better outcomes and are more satisfied with their care [36, 37]. Given the relationship between shared decision-making and evidence-based practice is becoming increasingly recognised, strategies to support clinicians in enacting a more patient-centred approach are required.

While this study has provided valuable insight into factors influencing surgeons’ decision-making around postoperative feeding, it has several limitations. It is possible that some views were not represented in our sample, however we used purposive sampling and conducted interviews across two sites to improve generalisability and continued data collection until saturation was reached, which may increase the relevance of our findings for similar settings.

## Conclusion

In summary, individual beliefs, patient-related factors and social influence of peers (particularly seniors) appeared to strongly influence surgeons’ decision-making regrading dietary prescription after colorectal surgery. As such, a multi-faceted approach to behavior change is required that targets each of these areas and is specific to the clinical context.

## Additional files

Additional file 1: Semi-structured interview questions based off the Theoretical Domains Framework (TDF). An example of the questions asked during interviews with staff. (DOCX 18 kb)

Additional file 2: Medical career structure. An outline of the Australian medical career structure. (DOCX 14 kb)

## Abbreviations

COREQ: Consolidated Criteria for Reporting Qualitative Research
EOF: Early oral feeding; ERAS: Enhanced Recovery After Surgery
NGT: Nasogastric tube
PEM: Protein-energy malnutrition
POD: Postoperative day one
S1: Site one
S2: Site two
TDF: Theoretical Domains Framework
