# Supplementary material for: A qualitative exploration of factors influencing medical staffs’ decision-making around nutrition prescription after colorectal surgery
Source: BMC Health Serv Res. 2019 Mar 19;19:178. doi: 10.1186/s12913-019-4011-7 (PMC6425714; doi:10.1186/s12913-019-4011-7)
Supplement: Supplementary file 1 — Semi-structured interview questions based off the Theoretical Domains Framework (TDF). An example of the questions asked during interviews with staff. (DOCX 18 kb) [file 12913_2019_4011_MOESM1_ESM.docx]

| **Additional file 1.** Semi-structured interview questions based off the Theoretical Domains Framework (TDF)^22^ | |
| --- | --- |
| **Domain** | **Core questions** |
| Knowledge | What do you know about recommencing feeding after surgery?  What is your understanding of the guidelines around recommence feeding after surgery? |
| Motivation and goals | What do you perceive to be the importance of nutrition after surgery?  What do you perceive to be the importance of feeding patients after surgery? |
| Professional role & identity | What is your role in providing postoperative nutritional care to patients (if any)?  Whose role is it to decide when a patient’s diet should be changed? |
| Emotion | Does anyone you work with have particularly strong feelings about feeding after surgery (either positive or negative)?  Does anyone you work with have particularly strong feelings about when patients should recommence solid foods after surgery (either positive or negative)? |
| Social influences | What do your peers think about postoperative feeding, and how it is enacted on this ward?  Is there anyone on the ward whose views or behaviours influence how you think about postoperative feeding? |
| Behavioural regulation | When do you usually prescribe feeding after surgery?  What diets types do you usually prescribe after surgery?  In what scenarios, if any, would you alter your usual feeding prescription practices?  What factors do you consider when deciding to upgrade a patients’ diet? |
| Beliefs about consequences | If any, what are the benefits of recommencing nutrition within 24h after surgery?  I any, what are the disadvantages if patients do not receive nutrition within 24h after surgery? |
| Skills | How confident do you feel in your ability to judge when a patient is ready to re-commence feeding after surgery?  If you are not sure whether a patient is ready to re-commence feeding after surgery, what do you do? |
| Environmental context and resources | Is there anything about your ward (or workplace) in particular that makes prescribing feeding easy or difficult?  When a patient’s diet code is upgraded, can you tell me how this should be conveyed through the treating team? |
| Beliefs about capabilities | Do you think patients should be involved in decisions around when and what they can eat after surgery? |
| Memory, attention, decision processes | In your practice, who is the major decision maker when it comes to upgrading patient diets? |
|  | |
