# Supplementary material for: A qualitative exploration of factors influencing medical staffs’ decision-making around nutrition prescription after colorectal surgery
Source: BMC Health Serv Res. 2019 Mar 19;19:178. doi: 10.1186/s12913-019-4011-7 (PMC6425714; doi:10.1186/s12913-019-4011-7)
Supplement: Supplementary file 2 — Medical career structure. An outline of the Australian medical career structure. (DOCX 14 kb) [file 12913_2019_4011_MOESM2_ESM.docx]

| **Additional file 2.** Medical career structure | |
| --- | --- |
| **Position** | **Description** |
| Intern | Doctors who have been accepted into an intern training program under the supervision of their employing hospital. Generally, this will be the 1st year of practice following completion of a medical degree. |
| Junior House Officer | Doctors in their first year of service after eligibility for full registration as a medical practitioner. Generally, this will be their second postgraduate year. |
| Senior House Officer | Doctors in their third or subsequent years of practical experience after eligibility for full registration as a medical practitioner. |
| Principal House Officer | Doctors in their fourth or subsequent year of postgraduate study. They are not undertaking an accredited course of study leading to a higher medical qualification. This position is an equivalent level to Registrar. |
| Registrar | Doctors undertaking an accredited course of study leading to a higher medical qualification. |
| Senior Registrar | Doctors who have a specialist registration with the Medical Board of Australia and are undertaking an accredited course of study leading to a higher medical qualification. |
| Fellow | Doctors who have completed their advanced training in a specialized filed and are required to do a Fellowship Year before they can get specialist registration (a requirement of some Colleges). |
| Consultant | The most senior doctors who are directly responsible for decision making related to hospitals and giving treatment to the patients. They have completed all of their specialist training and have been placed on the specialist register in their chosen specialty. |
